# Mapping Individual Brain Networks Using Statistical Similarity in Regional Morphology from MRI

**DOI:** 10.1371/journal.pone.0141840

**Published:** 2015-11-04

**Authors:** Xiang-zhen Kong, Zhaoguo Liu, Lijie Huang, Xu Wang, Zetian Yang, Guangfu Zhou, Zonglei Zhen, Jia Liu

**Affiliations:** 1 State Key Laboratory of Cognitive Neuroscience and Learning & IDG/McGovern Institute for Brain Research, Beijing Normal University, Beijing, 100875, China; 2 School of Psychology, Beijing Normal University, Beijing, 100875, China; 3 Center for Collaboration and Innovation in Brain and Learning Sciences, Beijing Normal University, Beijing, 100875, China; Chinese Academy of Sciences, CHINA

## Abstract

Representing brain morphology as a network has the advantage that the regional morphology of ‘isolated’ structures can be described statistically based on graph theory. However, very few studies have investigated brain morphology from the holistic perspective of complex networks, particularly in individual brains. We proposed a new network framework for individual brain morphology. Technically, in the new network, nodes are defined as regions based on a brain atlas, and edges are estimated using our newly-developed inter-regional relation measure based on regional morphological distributions. This implementation allows nodes in the brain network to be functionally/anatomically homogeneous but different with respect to shape and size. We first demonstrated the new network framework in a healthy sample. Thereafter, we studied the graph-theoretical properties of the networks obtained and compared the results with previous morphological, anatomical, and functional networks. The robustness of the method was assessed via measurement of the reliability of the network metrics using a test-retest dataset. Finally, to illustrate potential applications, the networks were used to measure age-related changes in commonly used network metrics. Results suggest that the proposed method could provide a concise description of brain organization at a network level and be used to investigate interindividual variability in brain morphology from the perspective of complex networks. Furthermore, the method could open a new window into modeling the complexly distributed brain and facilitate the emerging field of human connectomics.

## Introduction

Most studies examining brain morphology have focused on local morphological features with either voxel/vertex- or region-based methods [1]. As these local features are believed to reflect clinical conditions and individual differences in various tasks [2], they have become particularly popular as a neuroscience research topic. However, the human brain is a complex network that generates and integrates information from multiple sources [3]. In the past decade, researchers revealed some of the underlying architecture of nontrivial entities based on anatomical (e.g., through diffusion magnetic resonance imaging, dMRI) or functional networks (e.g., through functional MRI) [4]. Given these findings, it was expected that representing the brain morphology as a network would provide a holistic perspective on the understanding of the morphology of ‘isolated’ brain structures. For instance, with the network representation, researchers can obtain exclusive information reflecting an important aspect of the associations and interactions between multiple regions, which is not evident in local MRI information.

Thus far, several attempts have been made to construct morphological networks. Some researchers have proposed an approach involving estimation of interregional relationships, with covariance between averaged regional cortical thickness or gray matter (GM) volume measures across participants [5, 6]. However, this method can only construct one network with a large population and is incapable of constructing an individual morphological network. This limits its application in the investigation of individual variability in brain structure, particularly in identifying structural brain abnormalities in single patients. Comparing morphological features of different regions within individuals requires definition of correspondence mapping between the voxels of these regions. More recently, as a solution, Tijms et al. (2012) proposed an insightful strategy for constructing an individual morphological network. In such an approach, the entire brain is parcellated into 3 × 3 × 3 cubes (i.e., 27 voxels), and the pattern correlation between two cubes is defined as a connection. Although this approach was designed to capture the complex morphological structure of the brain, it does not take the remarkable variability in the shapes and sizes of different regions. More importantly, with such an approach, rigid extraction of the small cubes would not allow optimal correspondence with functionally or anatomically homogeneous regions of the brain [7].

To overcome the limitations mentioned above, we proposed a new network framework for individual brain morphology based on regional morphological distribution information. Technically, in the new network, nodes are defined as regions based on a brain atlas, and edges are estimated using our newly-developed interregional relation measure based on regional morphological distributions [8]. The connection metric allows estimation of the relationship between a pair of regions with different shapes and sizes and has been found to be informative for investigating individual brain morphology with MRI scans [8]. A similar distribution-based approach has been proposed to combine cortical surface and its connecting white-matter geometry to successfully characterize individual differences in brain structure [9]. Considering these strengths, we extended our initial investigation of single morphological connections and introduced the connection metric to the research field of complex brain networks. One of the competitive advantages of the new network framework is that it allows for the construction of brain networks for a single participant, using his or her MRI scans. Thus, the method has the potential to provide a new avenue for investigating intra- and interindividual differences in brain morphology at the network level.

In this study, we initially demonstrated our method in a sample of 21 healthy participants. Thereafter, we studied the graph-theoretical properties of the networks obtained and compared the results with previous morphological, anatomical, and functional networks. The robustness of the method was then assessed by measuring the reliability of the network metrics (N = 21, scanned twice). Finally, to illustrate potential applications, the networks were used to measure age-related changes in commonly used network metrics.

## Materials and Methods

### Participants

We used the Kirby21 dataset [10], consisting of 21 healthy adult volunteers with no history of neurological conditions (age range: 22–61 years, 10 females). Further details regarding the dataset can be found below and in Landman et al. (2011). The dataset is publicly available from Neuroimaging Informatics Tools and Resources Clearinghouse (https://www.nitrc.org/) and this study was approved by the Institutional Review Board of Beijing Normal University.

### Data acquisition

Structural MRI data were all acquired via the same 3.0T scanner (Achieva, Philips Medical systems) using a high-resolution 3D magnetization-prepared rapid acquisition of gradient echoes (MPRAGE; [11]) sequence with the following parameters: resolution, 1.0 × 1.0 × 1.2 mm; TR: 6.7 ms, TE: 3.1 ms, TI: 842 ms; flip angle: 8°; and SENSE factor: 2. High-resolution, high-contrast T1-weighted MRI data can be used for GM segmentation and quantification. Two structural MRI scans were performed for each participant (session 1 and session 2) on the same day, using the same protocol. For the sake of simplicity, demonstration of the method only involved the dataset from session 1, and the test-retest reliability estimation involved data from both sessions.

### Measure of regional GM volume

The MRI data were preprocessed via voxel-based morphometry (VBM) using Statistical Parametric Mapping, version 8 (SPM8, http://www.fil.ion.ucl.ac.uk/spm/). VBM is an automatic whole-brain neuroimaging analysis technique that allows the quantification of GM volume in individual MRI data. The analysis was conducted in a routine procedure. Specifically, MRI data for each participant was first checked manually by two experienced experts to ensure that there were no scanning artifacts. Second, GM images were obtained by segmenting individual MRI data followed by resampling to 2-mm isotropic voxels. This segmentation combines voxel intensity and prior probability maps for GM, white matter (WM), and cerebrospinal fluid (CSF) to make an initial probability estimate of a tissue type that a voxel most likely belongs to with a mixture model. The segmentation step also incorporates correction for intensity non-uniformity to account for non-uniformity artifacts [1, 12]. Third, the GM images were nonlinearly coregistered using Diffeomorphic Anatomical Registration Through Exponentiated Lie Algebra (DARTEL), which involved iterative calculation of a study-specific template based on the GM images from all participants and warping all participants’ GM images into the generated template. The study-specific template is used to enhance the estimation of local gray matter volume and reduce the registration error [13, 14]. Then, to make GM images in the same space as the brain parcellation (see below for details), the resulting GM images were then normalized to standard Montreal Neurological Institute (MNI) space. Thereafter, to preserve tissue volume following warping, voxel values in individual GM images were modulated by multiplying the Jacobian determinants derived from the normalization. Finally, all modulated GM images were smoothed individually with an 8-mm full-width at half-maximum (FWHM) Gaussian kernel. The spatial smoothing was applied to improve the signal to noise ratio, which helps improving the estimation of genuine GM volume. In sum, this is the standard processing procedure for estimating regional GM volume in VBM. This GM volume measure has been widely used to investigate the individual differences in local brain structure (e.g., [2]) as well as the hierarchical organization of human cortical network (e.g., [15]). The smoothed and modulated GM images consisting of the morphological information for each voxel, which was comparable across participants, were used in further analyses.

### Network construction from individual GM images

Construction of the connection matrix is a key issue in characterizing the human brain as a complex network [16]. To address this issue, the nodes and their connections should be determined first.

#### Node definition

Herein, nodes represent brain regions. To illustrate our approach, we used 90 (45 for each hemisphere) cortical and subcortical regions of interest (ROIs) from the automated anatomical labeling (AAL) atlas [17] as nodes. White-matter voxels in the raw AAL mask were removed if no gray-matter voxels existed within 2-mm cubic neighborhood based on the refinement procedure from previous studies [18, 19]. The AAL atlas has been widely used in previous brain network construction.

#### Edge definition

In the new network framework, we defined edges/connections (referred to as morphological connections) between different regions as the statistical similarity between their morphological measure distributions, using our newly-proposed method [8]. The connection was measured based on the symmetric Kullback-Leibler (KL) divergence measure as follows:

$$KL\left(p,\;q\right)=\int_{X}^{}\left(p\left(x\right)\text{log}\frac{p\left(x\right)}{q\left(x\right)}+q\left(x\right)\text{log}\frac{q\left(x\right)}{p\left(x\right)}\right)dx\;$$

where p and q are two distributions.

KL divergence was converted to a similarity measure using the following expression:

$$KLS\left(p,q\right)=e^{-KL\left(p,q\right)}$$

The KL-based similarity (KLS) ranges from 0 to 1, where 1 is for two identical distributions.

**Fig 1:**
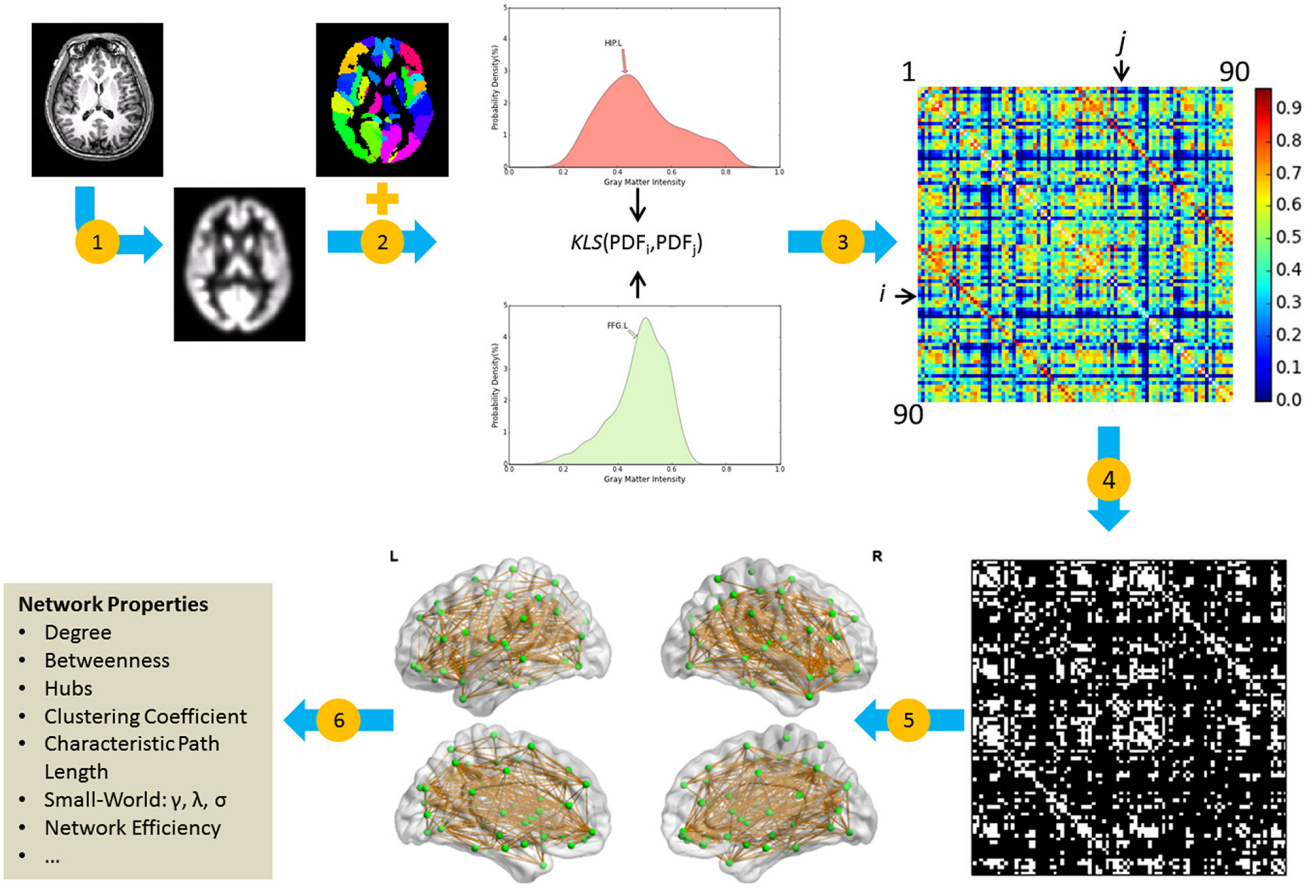
General workflow for the construction of an individual morphological network using gray matter measurements from MRI. (1) The estimation of gray matter volume as a morphological measure using a routine VBM procedure. (2) Brain parcellation with the AAL atlas and the estimation of morphological distribution for each region. (3) Repeated quantification of the similarity between morphological distributions for pairs of regions and formation of the similarity matrix by filling in corresponding similarity values. (4) Extract the binarized matrix at a specific sparsity threshold. (5) Represent individual brain network as a graph. (6) Calculate the network metrics (e.g., γ, λ, and σ). Note that, in this study steps 4–6 were repeated across a range of different sparsity thresholds, from 10% to 40% with an interval of 1%. HIP: Hippocampus; FFG: Fusiform gyrus; L: left; R: right; KLS: Kullback-Leibler divergence-based similarity; MRI: magnetic resonance imaging; VBM: voxel-based morphometry; AAL: automated anatomical labeling.

To quantify the connection matrices for individual networks, we estimated probability density functions (PDFs) for each ROI using kernel density estimation (KDE) [20] from the GM intensities of all voxels within the region (Step 2 in Fig 1). Specifically, the *Gaussian_kde* function implemented in the SciPy package (http://www.scipy.org/) was used. Kernel width was not set manually but was adaptively estimated from the data using Scott’s rule [21]. We then calculated the KLS values between all possible pairs of brain regions, resulting in a 90 × 90 similarity matrix for each subject (Steps 3 in Fig 1). Finally, individual similarity matrices were converted into binarized matrices (i.e., adjacency matrices), A = [*a*_*ij*_], according to a predefined threshold (see below for the threshold selection), where the entry, a_*ij*_, was 0 unless the similarity value between regions i and j was larger than the threshold, in which case it was 1 (Step 4 in Fig 1).

To provide a direct demonstration of the morphological network, an example brain network was visualized in the bottom-middle panel of Fig 1 with BrainNet Viewer [22]. In addition, we calculated the averaged network across the participants and the coefficient of variation (CV) map to show the consistency of the connections in the network across participants. CV, as a standardized measure of dispersion of quantity of interest, is defined as the ratio of the standard deviation to the mean.

### Network analysis

#### Threshold selection

Although continuous weights contain more information between nodes [23, 24], the present study assessed only the basic network topology, and therefore, the networks were binarized. More importantly, the binarized network allowed direct comparison with previous brain network studies (e.g., [7, 15, 18, 25]). To binarize the network, a sparsity threshold was used for all similarity matrices. Sparsity was defined as the ratio of the number of existing edges divided by the maximum possible number of edges in a network. This approach ensured that all similarity networks included the same number of nodes and edges by applying a subject-specific threshold and was used in the estimation of morphological connectivity [7, 26, 27]. Further, given the absence of a definitive method with which to select a single threshold, we binarized each interregional similarity matrix repeatedly over a wide sparsity range (from 10% to 40% with an interval of 1%) [28]. The main analyses were conducted using each of the sparsity thresholds.

In addition, to investigate whether the present method produced networks with properties that were comparable to previous studies, we highlighted the comparisons at a predefined sparsity threshold of 23%. The main reason for this was to perform a direct comparison with results from a previous study [7], which also aimed to extract individual networks using MRI scans. Moreover, such a moderate constraint could optimize interregional similarity strengths and be biologically plausible [29].

#### Network metrics

We calculated both regional and global network metrics for brain networks at each sparsity threshold. The regional metric included the nodal centrality metric, betweenness centrality. Global metrics included 1) small-world parameters, including the clustering coefficient (*Cp*), characteristic path length (*Lp*), and small-worldness (σ) and 2) network efficiency involving global efficiency (*Eg*) and local efficiency (*Eloc*). These network metrics were calculated by using GRETNA toolbox [30].

Betweenness. Betweenness centrality (*B*_*i*_) for node *i* is defined as the numbers of shortest paths between any two nodes that pass through those nodes [31]. We measured normalized betweenness as *b*_*i*_ = *B*_*i*_/*meanBet*, where *meanBet* was the average betweenness of all nodes in the network. The betweenness values reflect the importance of the nodes within the entire network.

Clustering Coefficient (*Cp)*. The clustering coefficient (*Cp*_*i*_) of a node is equivalent to the ratio of the node’s direct neighbors, which are also neighbors to each other [32]. The clustering coefficient (*Cp*) for the network is the average clustering coefficient for all nodes. Therefore, on average, *Cp* reflects the prevalence of clustered connectivity around individual nodes (i.e., functional segregation).

Characteristic Path Length (*Lp*). The characteristic path length (*Lp*) of a network is defined as the average shortest path length between all pairs of nodes [32] and the most commonly used measure of functional integration.

Small-World Properties. The small-world model was originally proposed by Watts and Strogatz (1998). Small-world networks have higher clustering coefficient relative to random networks and display similar characteristic path lengths to those of a random graph [32]. Small-worldness (*σ*) are defined as the division of the normalized clustering coefficient, *Cp*/*Cp*_*rand*_ (i.e., *γ*), and the normalized characteristic path length, *Lp*/*Lp*_*rand*_ (i.e., *λ*) [33]. *Cp*_*rand*_ and *Lp*_*rand*_ are the network metrics for comparable random networks (averaged from 100 comparable random networks for each network) [34]. We generated these random networks using Maslov’s random rewiring algorithm [34], which preserves the same number of nodes, number of edges, and degree distribution as the real network.

Small-world networks are defined by a clustering coefficient (*Cp*), which is larger than *Cp*_*rand*_, or a normalized value for *γ* larger than 1 and characteristic path length (*Lp*) that is approximately the same order as that of a comparable random graph or normalized value for *λ* close to 1 [4]. In addition, the syncretic metric (σ) is higher than 1 in a small-world network.

Network Efficiency. Global efficiency (*E*_*g*_) has been considered a superior measure of integration [35]. This network metric is defined as the average inverse shortest path length [35], which is related to the classical characteristic path length (*Lp*).

Further, the clustering coefficient can be regarded as a measure of the local efficiency of information delivery in the direct neighbors of each node. The local efficiency (*E*_*loc*_) of the network is estimated by averaging local efficiencies for all nodes in the graph.

Spatial Distribution of Hubs within the Networks. Important brain regions (i.e., hubs) often interact with many other regions. In this study, we applied normalized betweenness (*b*_*i*_) to assess the importance of individual nodes, as it captures the node's influence on the information flow between other nodes in the network. To quantify similarity and uniqueness between individual networks, two metrics were defined based on the spatial distribution of nodal importance; for the participant *m*, similarity to other participants was defined as average similarity with others, while uniqueness was defined as 1 minus maximal similarity with others. Specifically, these metrics were defined as follows:

$$Similarity_{m}=\frac{\sum_{n\in N,\;n\neq m}corr\left(Bd_{m},Bd_{n}\right)}{N-1}$$

$$Uniqueness_{m}=1-\underset{n\in N,n\neq m}{\text{max}}corr\left(Bd_{m},Bd_{n}\right)$$

where *N* is the total number of participants and *Bd*_*m*_ is the betweenness of all nodes for participant *m*. The scores ranged from -1 to 1 and 0 to 2 for similarity and uniqueness, respectively. These scores were subsequently rescaled to the range [0, 1].

Finally, we identified hubs in the proposed network. Specifically, we first calculated the average of the normalized betweenness centrality for each node across all participants and then defined hubs as nodes with an averaged betweenness value higher than one SD above the corresponding mean value across all nodes. To visualize the results, the BrainNet Viewer [22] was used.

### Test-retest reliability

The scan-rescan dataset allowed us to quantify the test-retest reliability of the morphological networks for each participant. Intraclass correlation coefficients (ICCs) were calculated, as shown below [36]:

$$ICC=\frac{\delta_{between}^{2}}{\delta_{between}^{2}+\delta_{within}^{2}}$$

where the ICC is conceptualized as the ratio of between-subjects variance to total variance.

The ICC is a quantity between 0 and 1, where 1 indicates perfect reliability (that is, the with-participant variance $\delta_{within}^{2}$ is nearly 0). An ICC value above 0.75 is considered excellent. An ICC value ranging from 0.59 to 0.75 is considered good, and results between 0.40 and 0.58 are considered fair [37, 38]. In this study, the “irr” package (http://cran.r-project.org/web/packages/irr/index.html) was used to estimate ICC values for each network metric assessed using individual networks over a wide range of sparsity thresholds.

### Statistical analysis of individual differences in the morphological network

To demonstrate the usability of the morphological network proposed in this study, we illustrated our approach with an exploratory study involving age-related changes in the brain network. In this analysis, we focused on the commonly used network metrics described above including *meanBet*, *Lp*, *Lp*, *λ*, *γ*, *σ*, *Eg*, and *Eloc*. In the analysis with predefined sparsity (i.e., 23%; see *Threshold Selection*) used for display purposes in the study, a significant threshold of p < 0.05 (FDR corrected for multiple comparisons) was applied. In addition, to ensure that the findings were robust to data non-normality and to avoid the influence of possible outliers, Spearman’s rank-correlation coefficients were calculated for each correlation analysis. The same analyses were repeated over the range of sparsity threshold values from 10% to 40%. Given the exploratory nature of the analyses, uncorrected p values (p < 0.05) were used.

## Results

For the first time, we proposed a new network framework of the individual brain morphology to investigate the brain structure at the network level. The following analyses were conducted. First, we described the spatial pattern of the proposed morphological networks. Second, we examined the small-world properties of the networks. Then, the spatial distribution of hubs was investigated. Next, the robustness of our method was estimated. Finally, we conducted a correlation analysis to explore the age-related changes in the network metrics.

### Description of the spatial pattern of the network

**Fig 2:**
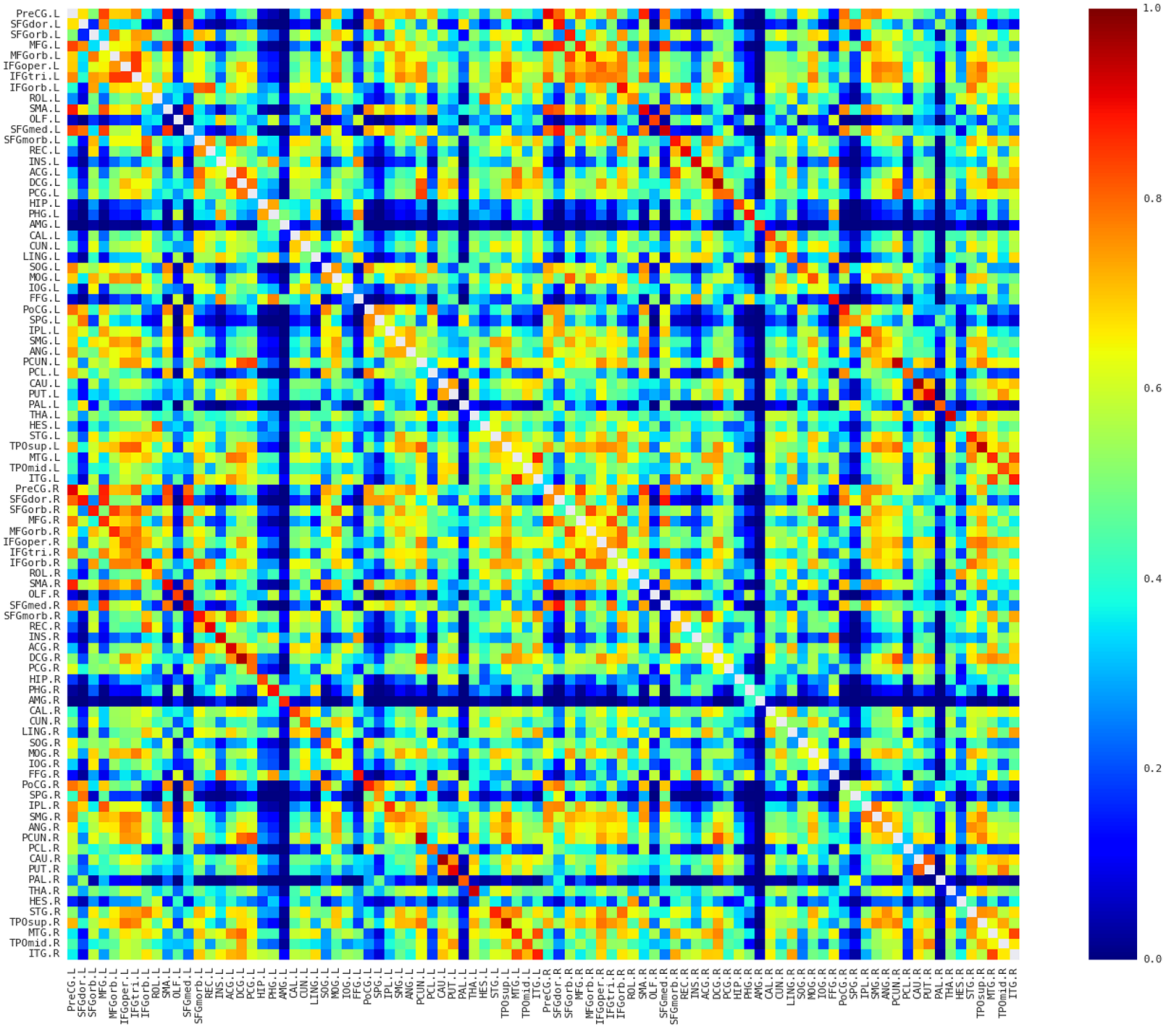
The averaged map of the connectivity matrices. Red and blue color indicates high and low similarity between regions, respectively. Main diagonal (i.e., self-connection) is indicated in white and excluded from following analyses. L, Left; R, Right.

**Fig 3:**
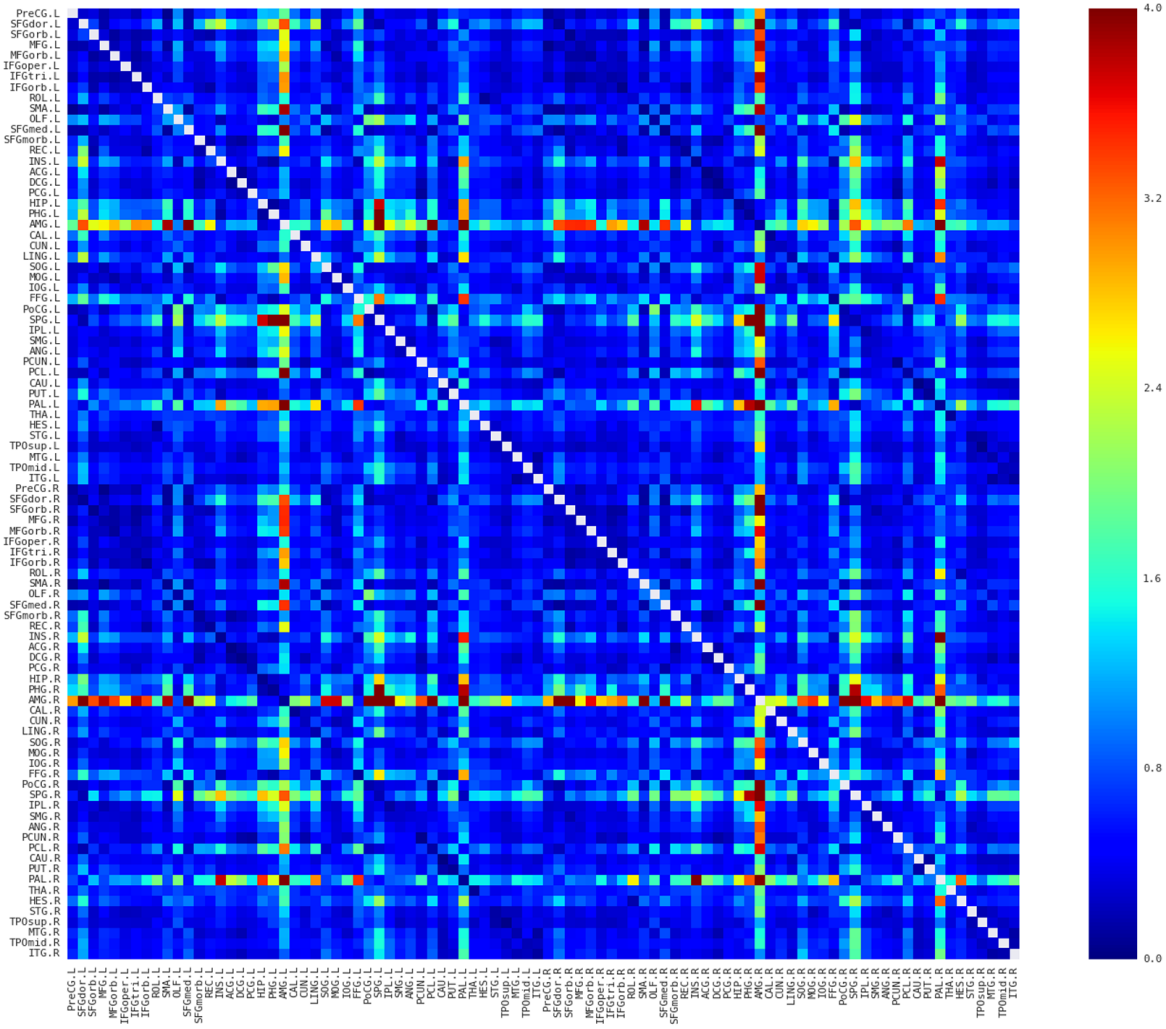
Coefficient of variation (CV) map of the connectivity matrices. Red and blue color indicates high and low dispersion of that connection across participants, respectively. Most of the connections possessed relative low CV and in particular the connections with relative high similarities showed low CV, suggesting relative high consistency across subjects. Main diagonal (i.e., self-connection) is indicated in white and excluded from following analyses. L, Left; R, Right.

The morphological networks were first constructed from MRI for each of participants with the proposed approach (see Materials and Methods; Fig 1). The individual network matrices were then averaged to reveal the similarity pattern of morphological distributions across participants. Several interesting facts can be observed in Fig 2. First, nearby regions showed similar connection pattern across the whole brain, such as STG, MTG, and ITG in the temporal regions, which is generally comparable with previous brain network studies (e.g., [39, 40]). Second, consistent with previous brain network studies (e.g., [39–42]), the homotopic regions from the two hemispheres showed high inter-hemispheric connections. Third, some intra-hemispheric regions located in different lobes also showed high similarity of morphological distributions. For instance, the relative high similarity between the frontal (e.g., PreCG and IFG regions) and partial (e.g., IPL and SMG) regions might reflect the white matter connection among these regions (i.e., superior longitudinal fasciculus). In addition, connections with relative high similarity showed relative low CV (top 5% connections: CV = 0.18; top 10%: CV = 0.22; top 20%: CV = 0.28; top 30%: CV = 0.31; Fig 3), suggesting considerable consistency across participants.

### Small-World properties of the individual networks

**Fig 4:**
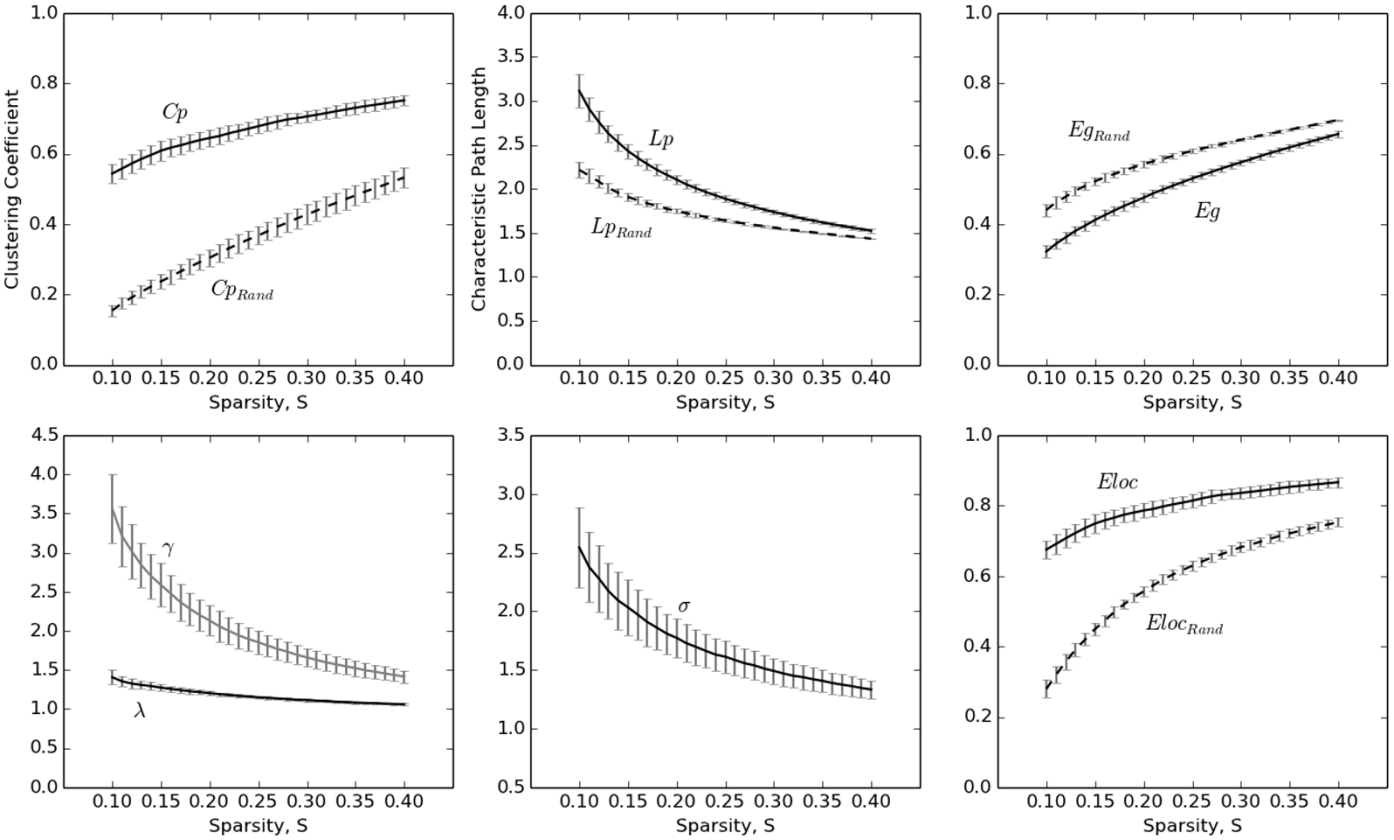
Small-world properties of the morphometric networks as a function of network sparsity thresholds. The error bar indicates the standard deviation.

Next, we assessed the small-world properties of individual morphological networks. Initially, their clustering coefficient (*Cp*) and characteristic path length (*Lp*) were compared with those from the comparable randomized networks. As expected, the clustering coefficients (*Cp*) for the extracted networks displayed significantly higher values, relative to those of the randomized networks, over wide sparsity thresholds (Fig 4, upper left; paired t test, range of t values: min = 44.706; max = 72.949; all ps < 8.502 × 10^−30^). Similarly, *γ* (i.e., *Cp/Cprand*) was larger than 1 (Fig 4, bottom left; range of average *γ* values: min = 1.416; max = 3.561), which satisfied the small-world network criterion. The characteristic path lengths (*Lp*) of the individual networks were also higher relative to those of the randomized networks (Fig 4, upper right; paired t-test, range of t values: min = 30.489; max = 61.904; all ps < 8.502 × 10^−30^). However, as shown in the bottom-left panel of Fig 4, the ratio of *λ* (i.e., *Lp/Lprand*) was close to 1 (range of average *λ* values: min = 1.062; max = 1.406), which also satisfied the small-world network criterion. Further, as expected, the small-worldness (*σ)* of these networks also displayed values larger than 1 (Fig 4, bottom right; range of the average *σ* values: min = 1.333; max = 2.543), supporting the small-world topology of the individual morphological networks extracted using our proposed method.

Previous morphological, anatomical, and functional network studies that reported network properties with similar network sizes within a predefined sparsity threshold (i.e., 23%, see Materials and Methods) in healthy individuals are summarized in Table 1. Another study [43] on individual morphological networks based on Tijms et al. (2012) shows similar global (Eg: ~0.53) and local efficiency (Eloc: ~0.77) as ours (Eg: 0.52; Eloc: 0.80) (Fig 4), though information on small-world properties such as γ and λ is not available for comparison. The results indicated that the small-world properties in this study were comparable to those of previous studies.

**Table 1:** Small-world properties in the present study, and for comparison, values from previous morphometric, anatomical, and functional studies.

Study	N	Cp	Lp	γ	λ	σ
**Morphological, Within Participant**						
Present study	90	0.66	1.92	1.74	1.15	1.50
Tijms et al. (2012) [7]	6982	0.53	1.86	1.35	1.05	1.28
**Morphological, Across Particiapnts**						
He et al. (2008) [28]	54	~0.30	~1.65	~1.35	~1.0	~1.3
Basset et al. (2008) [15]	104	~0.25	N/A	N/A	N/A	~1.25
Yao et al. (2010) [44]	90	~0.49	~1.89	~1.65	~1.10	~1.47
Zhu et al. (2010) [45]	90	~0.26	N/A	~1.2	~1.03	~1.17
Sanabira-Diaz et al. (2010) [46]	90	~0.31	~1.80	N/A	N/A	~1.25
**Anatomical**						
Gong et al. (2009) [18]	78	N/A	N/A	<3.0	~1.10	N/A
**Functional**						
Zhang et al. (2011) [47]	90	~0.33	~1.65	~1.30	~1.0	~1.4
Wang et al. (2009) [48]	90	~0.52	~1.75	~1.70	~1.05	N/A
Liu et al. (2008) [49]	90	~0.31	~1.77	~1.15	~1.01	~1.16

Note that the small-world properties from previous studies are all from healthy individuals. N: number of nodes used; N/A: Not Available.

### Spatial distribution of hubs within the networks

**Fig 5:**
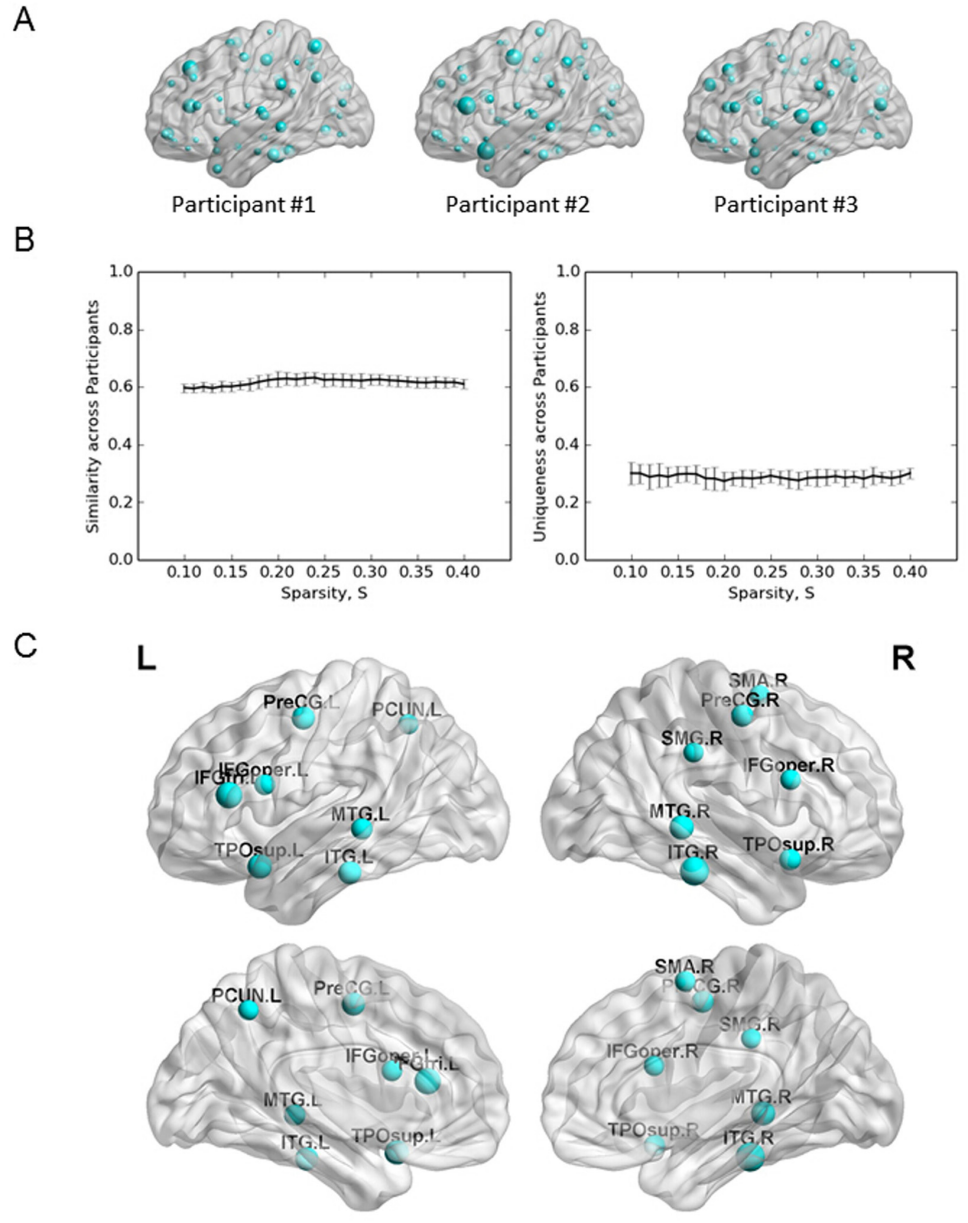
Spatial distribution of hubs within the morphometric network. (A) Three examples of the spatial distributions for betweenness. (B) The similarity (left) and uniqueness (right) of individual spatial distributions. (C) Hubs identified in this study. L: left; R: right. PreCG: precentral gyrus; IFGoper: inferior frontal gyrus opercularis; IFGtri: inferior frontal gyrus triangularis; SMA: supplementary motor area; SMG: supramarginal gyrus; PCUN: precuneus; TPOsup: superior temporal gyrus of temporal pole; MTG: middle temporal gyrus; ITG: inferior temporal gyrus.

In addition to the investigation of the small-world properties of the individual morphological networks, we also examined the spatial distribution of hubs (nodes with a normalized betweenness centrality higher than one SD above the mean for all nodes). Betweenness centrality is an important nodal metric that can be used to determine the relative importance of a node within the whole network and identify hubs in the complex networks, including the brain network (e.g., [18, 28]). Fig 5A shows the individual spatial distributions for 3 randomly selected participants, with larger spheres representing a higher betweenness value. The results visually showed that each individual exhibited a unique (though similar) spatial distribution. To give a quantitative description of the similarity and uniqueness of individual networks, we introduced two metrics respectively. Briefly, for one participant, the similarity to others is defined based on the average similarity with others, while the uniqueness is defined based on the value of 1 minus the maximal similarity with others. Results showed that individual networks possessed remarkable similarity (around 0.60; Fig 5B, left) as well as considerable uniqueness (around 0.3; Fig 5B, right) across different sparsity thresholds. The considerable uniqueness of individual networks may reflect the individual variability of brain anatomy [50, 51], while the remarkable similarity may reflect the consistent organization of human brain network (e.g., [16]).

Next, the hubs in the proposed network were identified (see Materials and Methods). Results showed that 14 hubs, including 10 heteromodal or unimodal associative regions, 2 regions of the primary motor cortex, and 2 paralimbic regions, were identified (Fig 5C; Table 2). All of these regions have been reported as hubs at least once in previous study (Table 2).

**Table 2:** Cortical regions identified as hubs in the morphometric network and their properties.

Hub Regions	Class	*Bet*	*Deg*	*Lp*	*Cp*	Previous morphological network studies
PreCG.L	Primary	1.98	27.45	1.76	0.64	m1, m2, m3, m6, a1, a2, f1, f5
PreCG.R	Primary	1.85	28.21	1.73	0.65	m1, m2, m3, m5, a1, f1
IFGoper.L	Association	1.70	31.88	1.63	0.64	m2, f3, f4
IFGoper.R	Association	1.65	30.24	1.64	0.65	m2, f3, f5
IFGtri.L	Association	2.42	34.48	1.57	0.61	m2, m4, f1, f4
SMA.R	Association	1.64	27.48	1.76	0.66	m4, m6, f1, f5
SMG.R	Association	1.58	30.38	1.66	0.67	m3, m6, f2, f5
PCUN.L	Association	1.62	31.48	1.62	0.67	M4, m5, m6, a1, a3, a4, f1, f2, f5
TPOsup.L	Paralimbic	2.15	32.64	1.60	0.65	M5, f2
TPOsup.R	Paralimbic	1.78	30.40	1.64	0.67	f2
MTG.L	Association	1.75	29.31	1.68	0.61	m2, m4, a3, f1, f2, f5
MTG.R	Association	2.12	29.67	1.68	0.61	m1, m2, m4, m5, a3, f1, f2, f5
ITG.L	Association	1.99	24.86	1.78	0.62	m1, m2, a4, f1, f2, f5
ITG.R	Association	2.77	27.79	1.74	0.58	m1, a4, f1, f2, f5

We compared the results with previous morphological network studies: m1 = [15], m2 = [52], m3 = [7], m4 = [27], m5 = [44] and m6 = [45]; anatomical network studies: a1 = [18], a2 = [19], a3 = [53], and a4 = [25]; and functional network studies: f1 = [54], f2 = [50], f3 = [55], f4 = [56], and f5 = [57].

Moreover, we revealed that the spatial distributions of hubs showed considerably more similarity (mean = 0.83, SD = 0.04) relative to its uniqueness (mean = 0.02, SD = 0.02) across different thresholds, suggesting that the observed spatial distribution of hubs is unlikely accounted for by any specific threshold.

### Test-retest reliability of the network metrics

**Fig 6:**
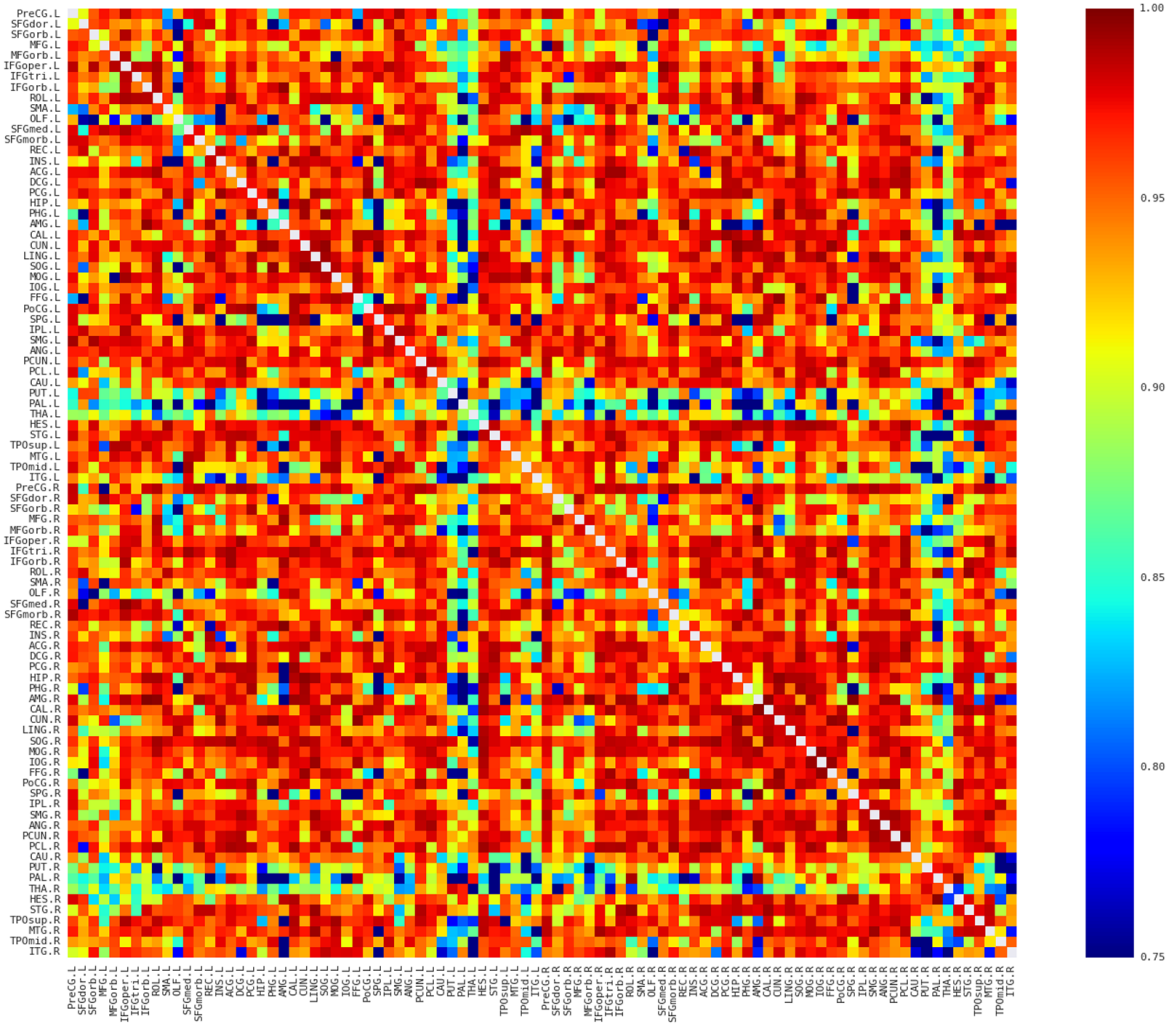
The Intraclass Correlation Coefficient (ICC) map of the connectivity matrices. More than 97% of the edges showed excellent reliability (i.e., ICC > 0.75).

To assess the robustness of our method, we estimated the ICC values for both the raw connections and each network metric with the test-retest MRI data (scanned twice). As shown in Fig 6, the connection values showed excellent reliability (mean ICC = 0.933, SD = 0.075) at the connection level; more than 97% of the edges possessed excellent reliability (i.e., ICC > 0.75), suggesting the high reliability of the proposed network.

**Fig 7:**
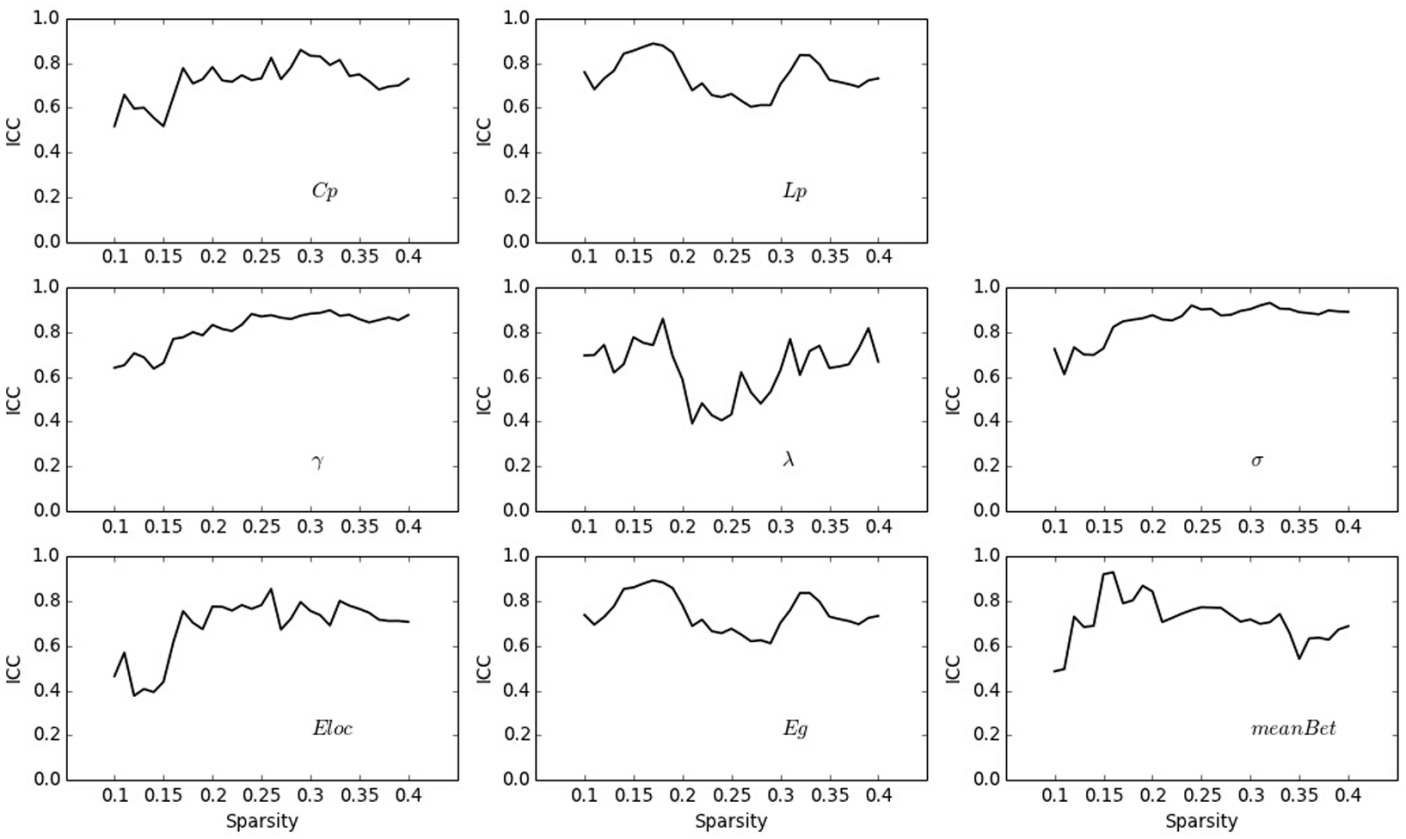
Test-retest reliability with intraclass correlation coefficient (ICC) for each of the network metrics as a function of network sparsity thresholds. The smallest ICC at various sparsity thresholds for each network metric is listed for the following: *Cp*: min ICC = 0.605, p = 0.0018; *Lp*: min ICC = 0.518, p = 0.0060; *γ*: min ICC = 0.636, p = 0.0020; *λ*: min ICC = 0.391, p = 0.035; *σ*: min ICC = 0.611, p = 0.00092; *Eloc*: min ICC = 0.377, p = 0.047; *Eg*: min ICC = 0.611, p = 0.0014; *meanBet*: min ICC = 0.485, p = 0.012.

As expected, the network metrics also showed considerable reliability (Fig 7). Under the predefined sparsity threshold value (23%), all commonly used network metrics displayed fair to excellent reliability (*Lp*: ICC = 0.657, p = 0.00044; *Cp*: ICC = 0.746, p = 2.09 × 10^−5^; *λ*: ICC = 0.428, p = 0.027; *γ*: ICC = 0.832, p = 7.24 × 10^−7^; *σ*: ICC = 0.871, p = 5.77 × 10^−8^; *Eloc*: ICC = 0.781, p = 7.34 × 10^−6^; *Eg*: ICC = 0.665, p = 0.00038; *meanBet*: ICC = 0.743, p = 2.47 × 10^−5^). Reliability analysis of these network metrics, assessed over a wide range of sparsity threshold values, indicated that the clustering coefficient (*Cp*) was highly stable (mean ICC = 0.740) for most of the sparsity values with which to threshold the morphological network, as were characteristic path length (*Lp*, mean ICC = 0.716), *γ* (mean ICC = 0.812), *σ* (mean ICC = 0.848), and global efficiency (*Eg*, mean ICC = 0.744). In addition, *λ* (mean ICC = 0.637), local efficiency (*Eloc*, mean ICC = 0.683), and betweenness centrality (*meanBet*, mean ICC = 0.717) were reproducible (see Table 3). Taken together, these results indicate the robustness of the proposed method for constructing individual morphological networks from gray matter MRI scans.

**Table 3:** A summary of test-retest reliability with intraclass correlation coefficient (ICC) for each of the network metrics.

Network Metric	ICC		
	Mean	Min	Max
***Cp***	0.74	0.605 (**)	0.887 (***)
***Lp***	0.716	0.518 (*)	0.859 (***)
***γ***	0.812	0.636 (**)	0.897 (***)
***λ***	0.637	0.391 (*)	0.859 (***)
***σ***	0.848	0.611 (**)	0.930 (***)
***Eloc***	0.683	0.377 (*)	0.853 (***)
***Eg***	0.744	0.611 (**)	0.892 (***)
***meanBet***	0.717	0.485 (*)	0.927 (***)

*: p < 0.05;

**: p < 0.005;

***: p < 0.0001.

### Applying the morphological network metrics to examine normal aging

**Fig 8:**
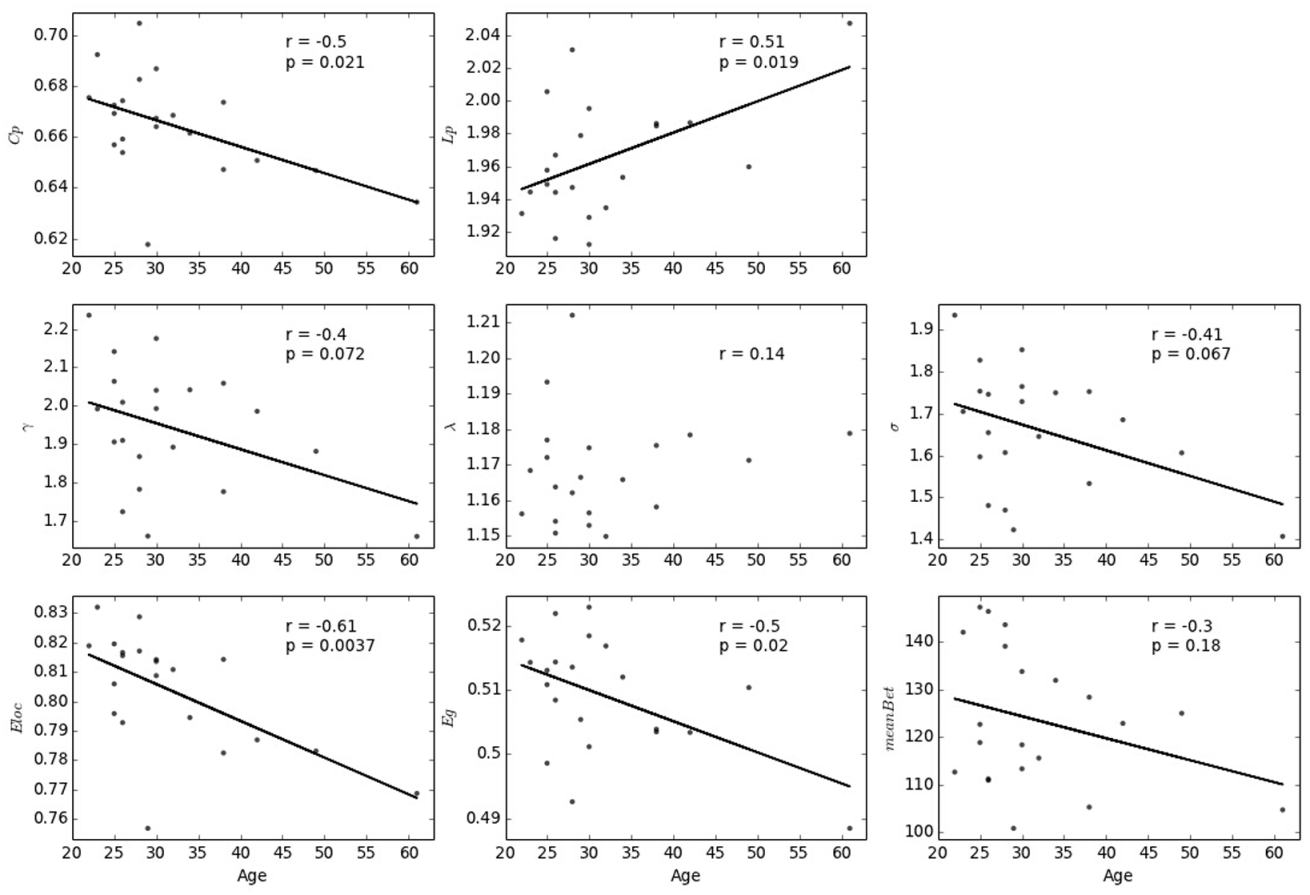
Age-related changes in each of the network metrics at a predefined sparsity threshold (i.e., 23%).

To demonstrate the usability of our method, we calculated the correlation between participant age and each of these network metrics assessed from individual networks. First, we conducted the analysis with a predefined threshold (i.e., 23%). As shown in Fig 8, significant age-related changes (p < 0.05, FDR corrected for multiple comparisons) were observed in the clustering coefficient (*Cp*, r = -0.50, p = 0.021; Spearman rho = -0.50, p = 0.021) and local efficiency (*Eloc*, r = -0.61, p = 0.0036; Spearman rho = -0.61, p = 0.0032). In addition, the effects of age on characteristic path length (*Lp*, r = 0.51, p = 0.019) and global efficiency (*Eg*, r = -0.50, p = 0.020) displayed notable trends but failed to reach statistical significance using Spearman’s rank-correlation (*Lp*: Spearman rho = 0.35, p = 0.12; *Eg*: Spearman rho = -0.35, p = 0.12). No significant age-related changes were observed in *γ* (r = -0.40, p = 0.072; Spearman rho = -0.34, p = 0.13), *σ* (r = -0.408, p = 0.067; Spearman rho = -0.30, p = 0.19), betweenness centrality (*meanBet*, r = -0.30, p = 0.18; Spearman rho = -0.22, p = 0.32), or *λ* (r = 0.14, p = 0.54; Spearman rho = 0.10, p = 0.64). Even with the most conservative correction approach for multiple comparisons (i.e., Bonferroni correction), the effects of age on the local efficient (*Eloc*) remained (p = 0.0036 < 0.05/8). The significant change in *Eloc* of individual morphological network indicates the age-related defect in local information delivery in the complex brain networks, which is in line with previous findings on white matter networks [19].

**Fig 9:**
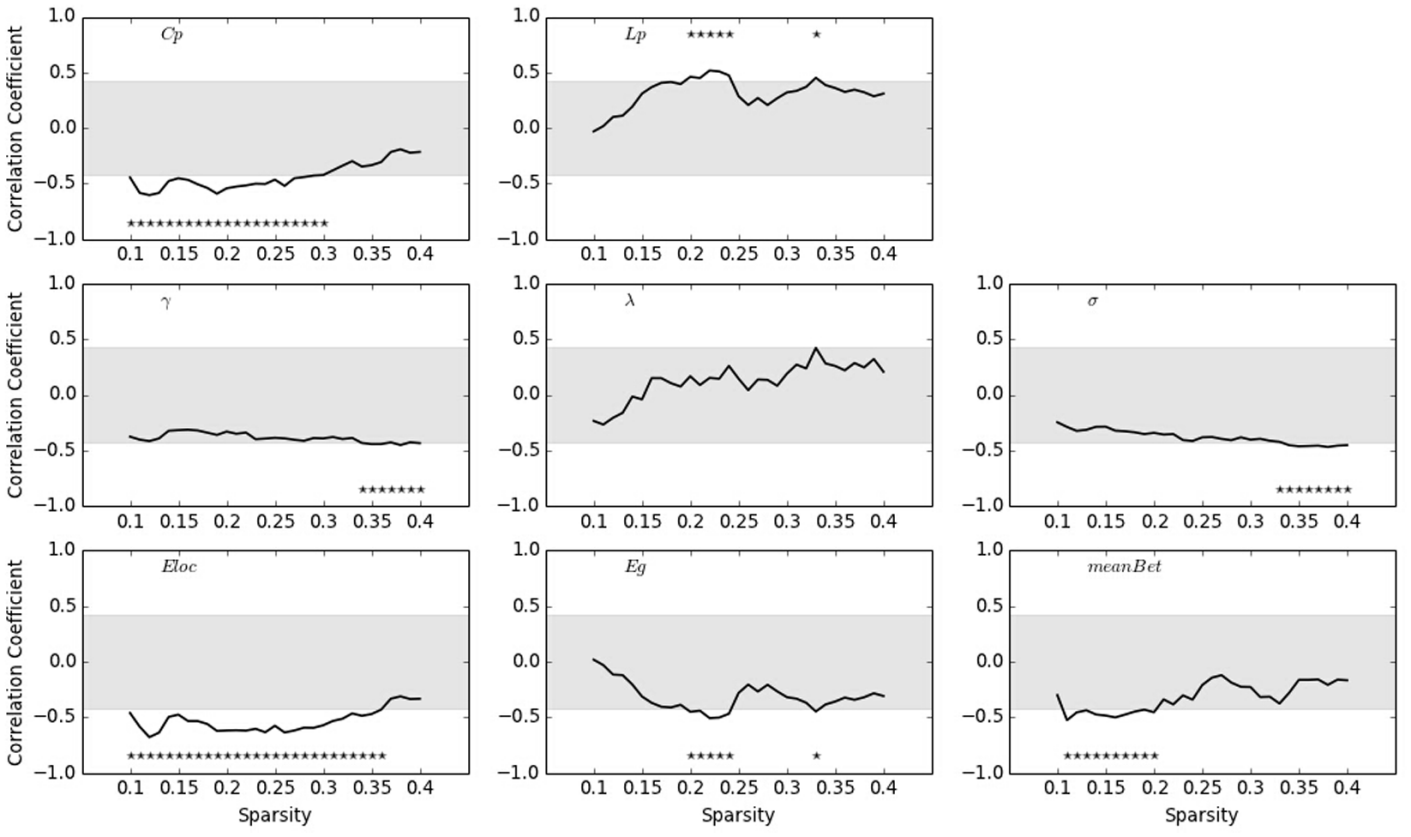
Age-related changes in each of the network metrics over the range of sparsity thresholds. The star markers (correlations falling outside the shaded area) indicate significant correlations (p < 0.05).

Results of analysis with a sparsity threshold value range of 10% to 40% are shown in Fig 9. Significant age-related changes (p < 0.05, uncorrected) in clustering coefficient (*Cp*) and local efficiency (*Eloc*) were observed for most of the sparsity values. In addition, the age-related changes in *σ* and *γ* were significant at higher sparsity values (S ≥ 33%). Characteristic path length (*Lp*), global efficiency (*Eg*), and normalized betweenness centrality (*meanBet*) also displayed significant age-related associations with some of the sparsity threshold values (*Lp*: 20% ≤ S ≤ 24%; *Eg*: 20% ≤ S ≤ 24%; *meanBet*: 11% ≤ S ≤ 20%). No significant associations were found observed with *λ* for any of the sparsity threshold values (max correlation value: r = 0.42, p = 0.057 with S = 33%).

In summary, our findings indicate the potential of the new approach to study the morphological networks of individual participants.

## Discussion

We proposed a novel network framework for individual brain morphology. The method was built on our recent work estimating interregional relation based on morphological distributions, by extending it into the research field of complex brain networks. The proposed method was able to construct and investigate brain networks for each participant, using MRI scans. With the use of graph-theoretical approaches, we found that all networks possessed small-world properties (i.e., they had a higher clustering coefficient relative to random networks and a similar minimum path length to that of comparable randomized networks). The values of the clustering and small-world coefficients were compatible with previous structural and functional network results (see Table 1). The morphological network also displayed hubs, all of which have been reported in previous network studies (see Table 2). In addition, the network metrics were reproducible, supporting the robustness of the method (see Table 3). Finally, the correlation analysis indicated that most of the network metrics reflected normal aging. Taken together, these results suggest that the new network could provide a concise description of the organization of an individual brain. Further, our approach provides a novel perspective on understanding of brain morphology and would further facilitate the emerging field of human connectomics.

### Inter-regional similarity of morphological distribution

It is noteworthy that, in this study, the inter-regional connection was quantified with similarity in distributions of local brain morphology. The inter-regional similarity metric has just been proposed recently and suggested to be informative for the study of brain morphology [8] However, it is almost entirely unknown whether this metric would provide benefits for mapping human whole-brain networks. The present study is, to our knowledge, the first to construct brain networks with regional morphological distribution information. In previous morphological network studies, networks have usually been constructed using correlations between average cortical areas, with respect to thickness or volume, across participants [15, 52, 58]. Another approach for mapping vertex-wise anatomical correlations has also been proposed [59]. However, such correlation-based approaches could only construct one network per group and required the analysis of networks at a large-group level.

In contrast, our method allows construction of individual brain networks using MRI images. More importantly, given that MRI images are widely available and convenient to collect, the proposed method could provide a novel perspective on the understanding of individual variability and clinical conditions. For example, this method could provide a more comprehensive means of automatically identifying brain abnormalities in a single participant’s brain. To construct individual networks based on MRI images, interesting methods have been used in particular contexts, which were based on average measures for different regions [60, 61] or even data from other participants [61]. Recently, Tijms et al. (2012) proposed an alternative method with which to extract individual networks using MRI scans of GM. Briefly, the method begins by defining approximately 6,982 nodes that correspond to 3 × 3 × 3 voxel cubes. The similarity between the nodes in the network is determined using correlation coefficients between two separate sets of 27 voxels from two cubes. This approach provides a new perspective on the examination of individual MRI data. However, in this approach, the rigid extraction of the small cubes may not allow optimal correspondence to functionally and anatomically homogeneous regions or convolutions of the brain [7], and under this framework, one has no choice but to define nodes as completely regular small regions with the same shape and size. Moreover, this approach can result in the problem of different network sizes for individual networks. This problem can be solved with a follow-up normalization strategy [43]. However, the cube size and rotation operation used in both studies are somehow arbitrary and does not take the remarkable variability in the shapes and sizes of regions into account. The present study proposed a new network framework based on regional morphological distribution information, which would provide a novel perspective for understanding individual differences and even clinical abnormality.

In addition, an interregional relationship was defined based on local brain morphology. The physiological meaning underlying the new measure is complex and not completely understood [8]. One possible source of the morphological connections proposed here is the axon tension theory [62], which predicts that connected areas are pulled by a mechanical force, becoming either thinner or thicker. This is similar to the population-based morphological relationship found in previous studies [15, 52]. An alternative possibility is that regions with similar morphological distributions could have reflected developmental coordination or synchronized maturation between areas, which may be related to axonal connections forming and reforming over the course of early development [59, 63]. Similarly, inter-regional relevance may also reflect common experience-related plasticity [64, 65]. Taken collectively, one could therefore speculate that individual morphological interregional relationships provide approximate reflection of true anatomical connections between neuronal elements. In addition, morphologically related regions may also share similar distributions of major cell classes (such as neurons, oligodendrocytes, and astrocytes) and gene expression [66]. Note that the abovementioned biological underpinnings are speculative, and future studies on cell morphology or molecular genetics are needed. Nevertheless, the novel method provides a reliable and meaningful description of the structure of individual brains. In addition, since different morphological metrics, such as GM volume, thickness, and area, measure different aspects of GM structure and thus can provide complementary information about local GM, future work is needed to extend our framework to accommodate multiple morphological features to measure the morphological similarity between regions.

### Spatial distribution of hubs

Previous studies have indicated that most of the hub regions, located in the association cortex [7, 18, 28, 59], play a central role in receiving convergent inputs from multiple brain regions [67]. With the present method, we found consistent spatial distribution. As expected, the hubs identified (Fig 3C; Table 2) in the morphological network were also predominately involved in the recently evolved heteromodal (e.g., the PCUN [precuneus], MTG [middle temporal gyrus], and IFGtri [inferior frontal gyrus triangularis]) or unimodal (e.g., the SMA [supplementary motor area] and ITG [inferior temporal gyrus]) association cortex [67]. As shown in Table 2, all hubs involved in this study have been reported as hub regions in several previous human brain network studies. For example, the PCUN and MTG have also consistently been identified as hubs in previous morphological (e.g., [27, 44]), anatomical (e.g., [53]), and functional (e.g., [54, 68]) network studies.

Of note, although there was overlap between the sets of hubs reported in these studies, which indicates a degree of topological isomorphism between different modal networks, no two studies have reported identical sets of hub regions. This discrepancy may have been caused by methodological differences, such as brain imaging parameters, connection and node definition, and the populations involved. Further systematic studies exploring hubs in brain networks from different modalities, with the same criteria of network analysis and in a similar population, would provide a deeper understanding of hubs in the human brain.

In addition, at an individual level, we found that participants showed unique (though similar) spatial distribution of betweenness values (Fig 3A and 3B). The considerable uniqueness observed may reflect the individual variability of brain anatomy [50, 51]. However, thus far, few studies have examined variability in hub distribution with anatomical, morphological, or functional brain networks. In response to this issue, we introduced two metrics to quantify the uniqueness and similarity of individual networks. We hope that further investigations into the source and outcome of this variability will benefit from these metrics.

Furthermore, as hubs have been identified as core regions with multimodal or integrative functions, their damage can affect the stability and efficiency of the network significantly [54, 69]. Studies conducted to determine whether patients with neurological diseases, particularly those with abnormality or deficit in the association cortex, have altered network attributes (e.g., small-world properties and modularity) in individual morphological networks are urgently required.

### Age effect on the network metrics

Age-related neuroanatomical changes are well recognized and believed to account for cognitive decline in normal aging [70]. Little is known about age-related changes in the organizational patterns of the whole-brain network. Here, network metrics estimated from each individual network were used to reveal the effects of age. Interestingly, significant reductions were found in local efficiency (*Eloc*) and the clustering coefficient (*Cp*), and an associative trend was found for global efficiency (*Eg*). Our results are highly compatible with previous findings. For instance, the significant change in *Eloc* in individual morphological networks is in line with previous findings on white matter networks, such as results of a study involving diffusion MRI that reported a significant effect of age on local efficiency, which was more pronounced than that on global efficiency [18]. There have also been inconsistent findings. For example, in a morphological network study across participants [27], although local efficiency was consistently found to be significantly larger in a young group (18–40 years) compared to middle-aged (41–60 years) and old (61–80 years) groups, the global efficiency of the young group was significantly lower relative to those of the middle-aged and old groups. Methodological differences (mainly the connection definition) may have been responsible for this discrepancy. In addition, the study population may also play a role, as in the present analysis, most of the participants were young (18/21 were aged 20–40 years) according to the criteria established by Wu et al. (2012). Further, considering the relatively small sample size and complicated (and non-linear) fashion in which network properties were computed, further systematic studies with larger cohorts and more flexible statistical models are required. Nonetheless, the present findings demonstrated potential for use in revealing presumed whole-brain network individual differences in healthy populations and abnormalities in participants with neurological and psychiatric disorders, as have been found in anatomical [71–73], functional [74–76], and population-based morphological networks [15, 26, 44].

### Methodological issues and future research

The present study was subject to some methodological issues that should be addressed.

First, we defined connections in the networks based on morphological distributions. Therefore, accurately estimation of such distributions is of critical importance. On one hand, there should be a sufficiently large sample from such a distribution. In the present study, the AAL atlas was used to obtain macroscopic parcellation of the brain, as in previous studies (e.g., [18]). For each region, there were sufficiently large observations (larger than 120), which ensured accurate estimation of regional morphological distributions. In addition, with increasing resolution, morphological networks could also be based on finer parcellation of the brain. On the other hand, in the present study, Gaussian KDE [20] was used to estimate morphological distributions. Although the present method was initially validated [8] and appears to produce good approximations, it would be interesting to examine how different estimation methods influence individual morphological networks.

Second, in the present study, we binarized each network repeatedly over a wide range of sparsity thresholds, as in previous morphological network studies [7, 26, 27]. This was performed in consideration of the absence of a definitive method for selecting a single threshold. Given continuous weights contain more information [23, 24], it is also possible to characterize the brain as weighted networks. However, the weighted models may lead to complicated statistical descriptions in the graph theoretical analyses [52, 75, 77], so we confined our analyses to a simple binary network analysis. It is worthy of applying graph theoretical methods to the weighted networks to investigate the topological properties of the individual morphological networks in future studies.

Third, the network analysis in the present study was based on the AAL template. As previous network studies with either resting-state fMRI or diffusion MRI have suggested that the measure of similarities likely depended on the choice of brain templates to some extent [48, 78], which is an inevitable limitation of any method on large-scale brain network. Future studies are needed to quantify whether and how different templates (e.g., the Harvard-Oxford atlas) affects network properties (e.g., small-world properties) of individual morphological network. In addition, it is a potential topic to construct and investigate the individual morphological networks with features, such as thickness, area and curvature, and brain parcellation derived in the native space.

Fourth, the test-retest reliability was performed based on the datasets collected in the same day to ruling out unwanted effects of training or experience. However, this was short-term reliability, and further studies are needed to examine long-term reliability for this method [79].

Finally, although the local GM volume was used to construct individual brain networks in the present study, other morphological (e.g., cortical thickness and area from Freesurfer, tissue density, and cell type) and non-morphological features (e.g., statistical parametric mapping in task fMRI) may also be appropriate. For example, our method could allow the construction and study of functional networks using statistical parameter maps [80], rather than original fMRI time series, which have been proven to be more vulnerable to confounding factors, such as in-scanner head motion [81–83]. In this case, researchers could explore the different network properties during the performance of different tasks. Similarly, these networks may also reflect individual differences in task performance. As another example, in constructing a brain network using the only ultrahigh-resolution brain model, BigBrain [84], the similarity of regional distribution in microscopic data may result in a concise and meaningful measure of connections at an almost cellular level. In all, it would be interesting to explore more detailed patterns using these local features from the perspective of complex networks.

## Conclusions

Complex network analysis has emerged as an important tool in the characterization of anatomical, functional, and morphological brain connectivity. We proposed a new framework for constructing individual morphological networks using MRI data with our newly-developed interregional relation metric based on regional morphological distributions. Results showed that the morphological networks possessed prominent small-world properties and spatial distribution of pivotal regions, mainly the association cortex regions. In addition, the network metrics displayed excellent reliability, which supports the robustness of the method. Our findings are largely compatible with previous human brain network studies. In summary, our method may provide a novel insight into the cerebral organization that underlie interindividual variability in behavioral and cognitive performance and clinical conditions. We hope that the brain mapping community will benefit from and contribute to this novel method.
